# Translaryngeal Tracheostomy Needle Introducer: a simple device to improve safety and reduce complications during Fantoni’s translaryngeal tracheostomy procedure: trial on human cadavers

**DOI:** 10.1186/s40635-019-0221-x

**Published:** 2019-01-28

**Authors:** Alessandro Terrani, Enrico Bassi, Caterina Valcarenghi, Emmanuel Charbonney, Paul Ouellet, Patrice Gosselin, Giacomo Bellani, Giuseppe Foti

**Affiliations:** 1 Department of Emergency and Intensive Care, ASST, Monza, Italy; 2 Desio Hospital, Via Mazzini 1, Desio, Italy; 3 Opendot Lab, Via Tertulliano 70, Milano, Italy; 4 University of Milan, Milan, Italy; 5 Centre de Recherche de l’Hôpital du Sacré-Coeur, Université de Montréal, Montreal, Canada; 6 Département d’anatomie, Université du Québec à Trois-Rivières, Trois-Rivières, Canada; 7 Cardiac Arrest and Ventilation International Association for Research (CAVIAR group), Edmundston, NB Canada; 8 Department of Surgery, University of Sherbrooke, Quebec, Canada; 9 Vitality Health Network, North West Zone, Edmundston, NB Canada; 10 Hôpital Pierre-Le Gardeur, Terrebonne, Canada; 11 School of Medicine and Surgery, University of Milan-Bicocca, Via Cadore 48, Monza, Italy

**Keywords:** Tracheostomy, Percutaneous, Translaryngeal tracheostomy, Needle introducer, Safety, Complications, Digital fabrication, 3D printing

## Abstract

**Background:**

Percutaneous dilatational tracheostomy (PDT) is the most frequently performed procedure in patients requiring prolonged mechanical ventilation. A crucial step in such procedures is needle insertion into the trachea. To simplify this procedure and increase its safety, we developed a new device, the translaryngeal Tracheostomy Needle Introducer (tTNI), for use with Fantoni’s method. This cadaver study was designed to assess the performance of the tTNI on human anatomy.

**Methods:**

We tested the tTNI in a cadaver laboratory; the operators included two experts trained in PDT and three without specific training in the procedure. We performed 58 needle insertion attempts on 13 cadavers. We compared the tTNI technique with the standard needle insertion approach using external landmarks. We recorded the number of attempts needed to optimise needle insertion, time required in seconds, final position of the needle and complications related to needle insertion.

**Results:**

tTNI use resulted in fewer puncture attempts (1.91 ± 1.34 vs. 1.19 ± 0.5, *p* < 0.001), less time (36.8 ± 51.6 s vs. 13.14 ± 15.57 s, *p* < 0,001) and increased precision on the first puncture (18.87 ± 25.38° vs. 7.5 ± 12.95°, *p* < 0,005). We did not observe any complication with tTNI use, whereas complications found using the standard method were in line with the literature.

**Conclusions:**

The tTNI is a device that simplifies needle insertion by enhancing the accuracy of insertion with fewer attempts and higher precision, even when used by less experienced operators. Clinical testing is required to evaluate the device performance in patients.

**Electronic supplementary material:**

The online version of this article (10.1186/s40635-019-0221-x) contains supplementary material, which is available to authorized users.

## Background

Percutaneous dilatational tracheostomy (PDT) techniques are frequently used in patients who need tracheostomy for prolonged periods of mechanical ventilation in intensive care units (ICUs) [1]. In 2015, approximately 6000 percutaneous and surgical tracheostomies were performed in Italy [2].

Several different PDT techniques and instruments have been developed with varying technical characteristics, including Ciaglia Blue Rhino [3], Percutwist [4], Fantoni’s translaryngeal method [5, 6], Griggs’s method [7] and Ciaglia Blue Dolphin [8]. Regardless of the technique, the procedure can be schematically divided into three sequential steps: tracheal needle insertion (common to all techniques), dilatation of the tracheal stoma over a guidewire with various devices depending on the specific technique and endotracheal cannula insertion or rotation. Most of the innovations have focused on the device used to open the tracheal stoma; however, there has yet been no easy method for ensuring safe needle insertion into the trachea. The most frequent method used to verify correct needle position within the trachea is through flexible bronchoscopy.

To date, the rate of complications associated with PDT remains high [9]. The most common complications related to needle insertion are bleeding, puncture of the endotracheal tube or endoscope, false passage of the guidewire or cannula, puncture of the posterior tracheal wall, oesophageal puncture, subcutaneous emphysema and pneumothorax [9]. The incidence of posterior tracheal wall puncture, one of the most dangerous complications, varies from 0.2 to 12.5% [10].

Different devices specifically developed for precise needle insertion into the trachea already exist [11–14]. However, none of the devices fulfilled all the safety standards, which, based on our experience, we deemed essential. These include a continuous endoscopic view of the trachea during the procedure, avoidance of a metal stylet in the tracheal lumen and prevention of a device from extending beyond the distal end of the endotracheal tube.

To create a device allowing the safe introduction of the needle into the trachea, we were inspired by the Stryker Gamma3 long nail system™ for femoral fractures [15]. An external mechanical arm (named Targeting Arm) is used to precisely guide the screws blocking the endo-medullary nail to within millimetres of the correct position. Following the same basic principle, we developed an external arm to guide tracheal needle insertion based on the rigidity and known measurements of the tracheoscope used for Fantoni’s procedure (Covidien-DAR™).

At our centre, Fantoni’s translaryngeal tracheostomy (TLT) [5, 6] is the procedure of first choice due to its intrinsic safety, especially in patients with increased bleeding risk (e.g. patients on anti-coagulation therapy for intra-aortic balloon pumps or extracorporeal membrane oxygenation, on antiplatelet therapy or with thrombocytopenia).

With the aim of simplifying tracheal needle insertion during TLT and reducing the number of attempts and related complications, we designed a novel device (pending Italian patent) named the translaryngeal Tracheostomy Needle Introducer (tTNI). In this study, we assessed the performance of the tTNI for accurate needle insertion using human cadavers.

The primary objective of the study is to verify the greater safety and precision of the tracheal puncture made with the tTNI compared with that made with the standard technique (freehand with anatomical landmarks) using cadavers.

The secondary objective of the study is to verify that tTNI use guarantees greater safety and precision of the tracheal puncture for trained and untrained operators.

## Methods

### Device conception and production

The tTNI was designed and tested, and a prototype was made using digital fabrication technologies (mainly laser cutting and 3D printing) in an Italian Fablab, OPENDOT [16], under the supervision of Dr. Enrico Bassi. The laboratory was chosen for its interest in the health care field and previous work done in collaboration with therapists and patients [17]. The 3D design of both parts was prepared using the Rhinoceros© software with the following anatomical parameters [18] entered: tracheal diameter, distance between the skin and anterior tracheal wall, prominence of the chin and distance between the tracheal rings.

**Fig. 1:**
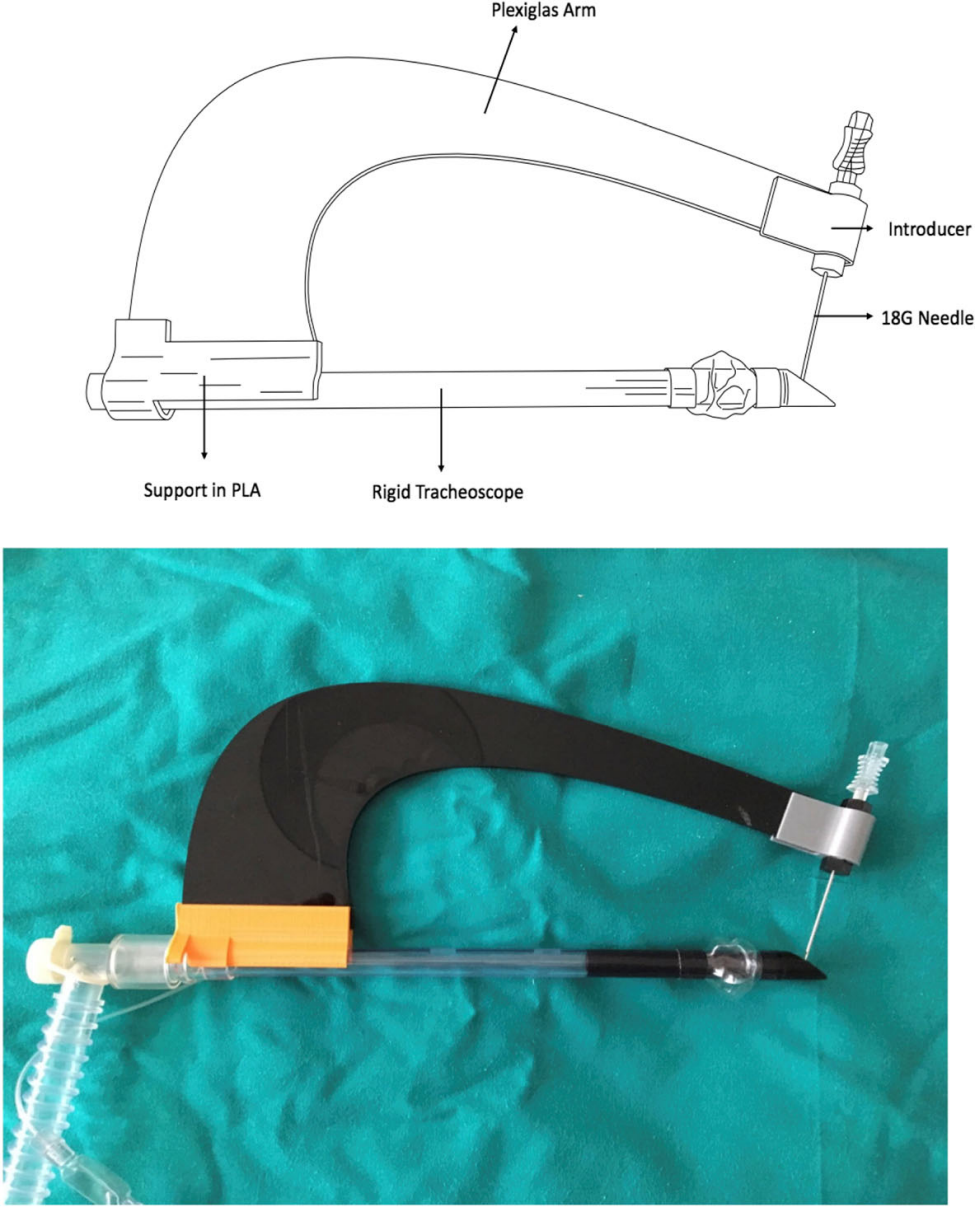
Technical design of the translaryngeal Tracheostomy Needle Introducer. PLA, polylactic acid

The shape of the device (Fig. 1) was designed to provide these specific features:

- The needle tip must be aligned with the centre of the tracheoscope.
- The inclination of the needle must allow for correct passage between the tracheal rings.
- The needle grip has a stopper against the rim of the introducer that prevents overshooting when the needle tip reaches the correct position in the centre of the tracheoscope lumen.
- The posterior tracheal wall is protected by the ‘flute beak’ of the rigid tracheoscope.

The final tTNI version (1.5) is illustrated in Fig. 1:

- Rigid tracheoscope (endotracheal tube, Fantoni’s kit: Covidien-DAR™, Segrate, Milan, Italia)
- Plastic support for the arm of the tTNI made with polylactic acid
- Shaped Plexiglas arm (generic acrylic structure)
- 18 G angiographic needle (7 cm)
- Introducer for the 18 G needle

An additional video shows the assembly of the device in more detail (see Additional file 1).


Additional file 1:Assembly of the device. (MP4 16003 kb)


Before experimenting on cadavers, we tested the prototypes on a mannequin used for teaching basic life support, which has artificial airways and trachea. The first version of the tTNI, made of plastic and plywood, was improved after extensive tests on the mannequin, resulting in the latest version.

## Ethics

The ethics board of the Anatomy Laboratory at the Université du Québec à Trois-Rivières approved this research (SCELERA-15-05-PR02, Research Anatomy Institutional Review Board sub-committee).

## Cadaver experiments

Thirteen cadavers used for the experiments were from persons who had donated their bodies for scientific research through the donation programme of the SIVA-CAVIAR Laboratory, University of Quebec, Anatomy Department, Trois-Rivières, Canada. They had been embalmed with the Thiel soft-fix embalming method, making them suitable for procedural research [19, 20].

Endoscopy and image recording were done using single-use bronchoscopes (Ambu® Scope™ 3, Ambu; Ballerup, Denmark).

**Fig. 2:**
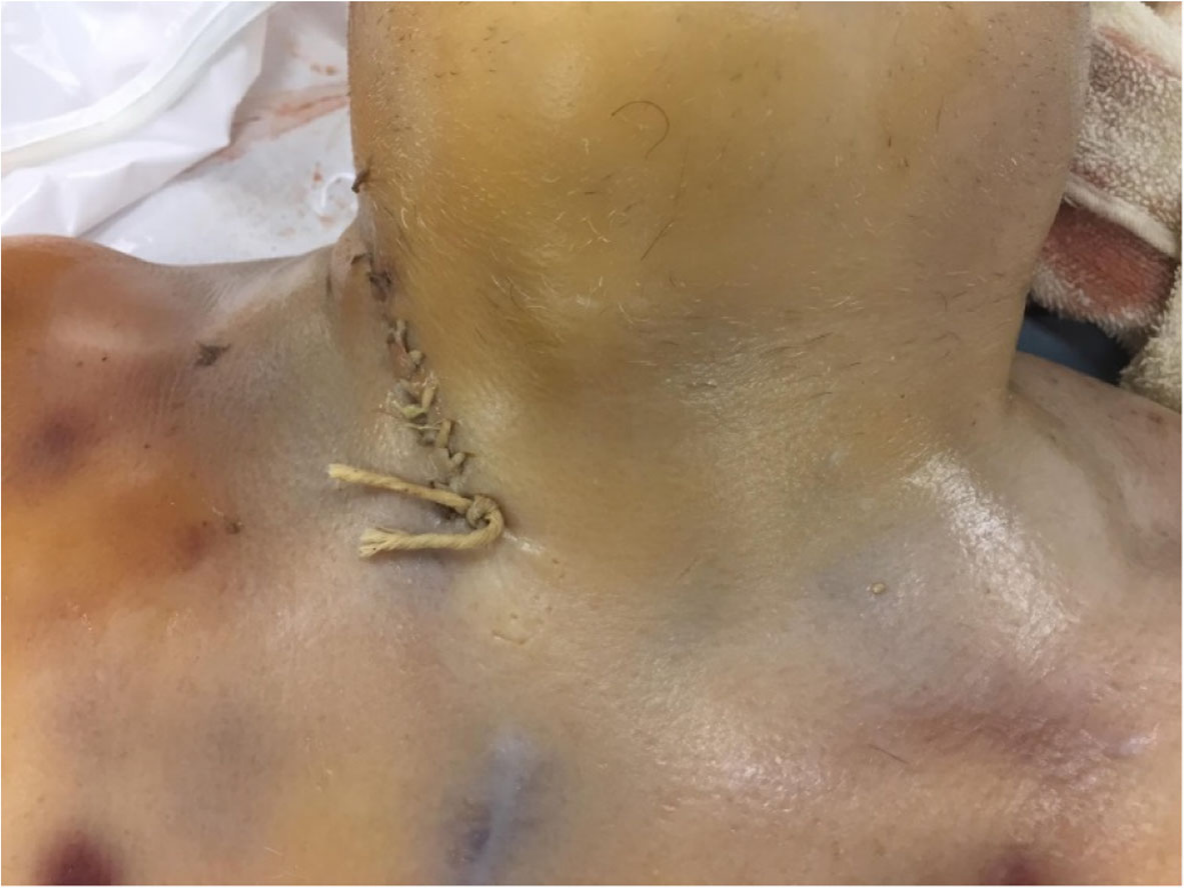
Even repeated needle punctures did not leave obvious marks that might affect the experiments

No make-up or covering was necessary between the first and successive punctures because previous punctures were not visible with the standard settings for the experiment (Fig. 2).

### Subject and training

The tracheostomy needle was introduced in the cadavers by five operators with varying skill levels in PDT. Two were trained physicians who had performed > 50 procedures and underwent regular practice in PDT. The other three were untrained, including one physician, one respiratory therapist without any experience in PDT and one physician who had not performed PDT for > 10 years. Other than an explanation of the parts of the tTNI, no formal training was given to any operator prior to performing the procedure.

## Experiments

### Description of the standard procedure and tTNI use

First, we inserted a flexible bronchoscope into the rigid tracheoscope and used a standard Macintosh laryngoscope to allow a clear direct view of the larynx. Then, under continuous endoscopic vision, the rigid tracheoscope was inserted into the trachea following the instruction of Fantoni et al. [5, 6] using a direct or a retro-molar space approach. Both the standard and tTNI techniques were performed using the rigid tracheoscope in the trachea (in ventilated patients in clinical practice, the first step of the standard technique consists of the replacement of the normal endotracheal tube with the rigid tracheoscope via tube exchange or reintubation under continuous endoscopic vision: needle insertion is performed through the identification of anatomical landmarks).

**Fig. 3:**
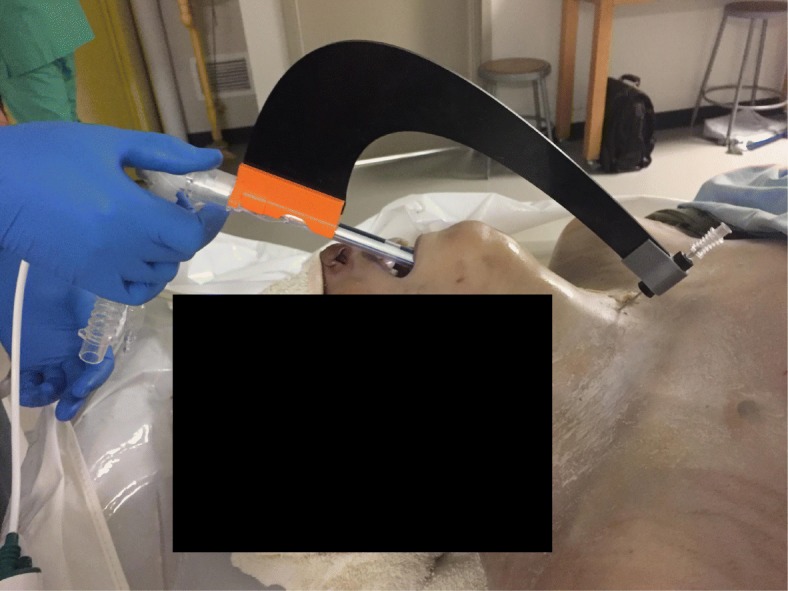
Needle insertion with translaryngeal Tracheostomy Needle Introducer. The rigid tracheoscope is inserted directly, and there is continuous endoscopic monitoring


Each operator used the standard approach with the external anatomic landmarks or the tTNI (Fig. 3) to perform needle insertion. The additional videos show tTNI use in more detail (Additional files 1, 2 and 3). The two trained physicians and two of the untrained operators attempted both procedures on all 13 cadavers; the remaining untrained operator made the attempts on six bodies. Both the sequence of operators and the use of the standard method or tTNI were randomised.

Additional file 2:Use of tTNI: example 1. (MP4 8700 kb)



Additional file 3:Use of tTNI: example 2. (MP4 19500 kb)


For each attempt, the following variables were recorded:

- Number of attempts to achieve optimal needle insertion (number of skin punctures + number of times the needle was redirected after skin puncture until optimal needle insertion). Optimal needle insertion was defined as the needle tip entering the tracheal lumen between − 30° and + 30° from the anterior tracheal wall midline (with the midline being 0°) and between the first and second tracheal rings.
- Time required in seconds to complete the procedure. The time from when the first neck puncture was made till correct position of the needle tip was achieved was considered, whereas the time required for the setup of the tTNI (about 10 s) was not considered. A maximum time of 300 s was allowed. The time was considered an index of simplicity but not of safety.
- Final position of the needle once the procedure was completed (number of degrees from the anterior tracheal wall midline).
- Adverse events and possible complications related to needle insertion that were detectable on the cadavers, defined as posterior tracheal wall puncture, lateral tracheal wall puncture, final needle position off-target, external puncture of the endotracheal tube or damage to the cuff of the tube.

## Statistical analysis

The non-parametric Wilcoxon signed-rank test was used to compare the number of attempts, time needed and precision of puncture among all operators. We used a chi-square test to compare the rate of complications between the tTNI and standard percutaneous method. A *p* value of < 0.05 was considered statistically significant.

## Results

The 13 cadavers had varied realistic anatomic features and proved to be suitable for the procedure (Table 1). The endoscopic images were equally accurate and realistic. Two of the cadavers had had the thorax opened and the lungs removed but had sufficient tracheal length for the procedure.

**Table 1:** Characteristics of cadavers used to test tracheal needle puncture techniques

Sex	Age (years)	Height (m)	Weight (kg)	BMI (m^2^)	Cormack–Lehane score (direct laryngeal vision)	Notes
Male	51	1.70	61.0	21.1	1	
Female	51	1.70	36.0	12.5	1	
Male	72	1.78	72.5	22.9	1	
Female	34	1.66	74.5	27.0	1	
Male	76	1.68	87.0	30.8	3	Short neck
Female	61	1.57	52.0	21.1	1	
Male	68	1.70	72.6	25.1	1	
Male	79	1.60	68.1	26.6	1	
Female	78	1.54	66.0	27.8	1	
Male	80	1.78	86.1	27.2	3	Short neck
Female	64	1.65	35.0	12.9	1	
Male	66	1.73	40.0	13.4	1	Lungs removed
Male	69	1.73	54.	18.0	1	Lungs removed

Cormack–Lehane Score: 1 easy intubation, 2 less easy, 3 difficult, 4 very difficult

**Fig. 4:**
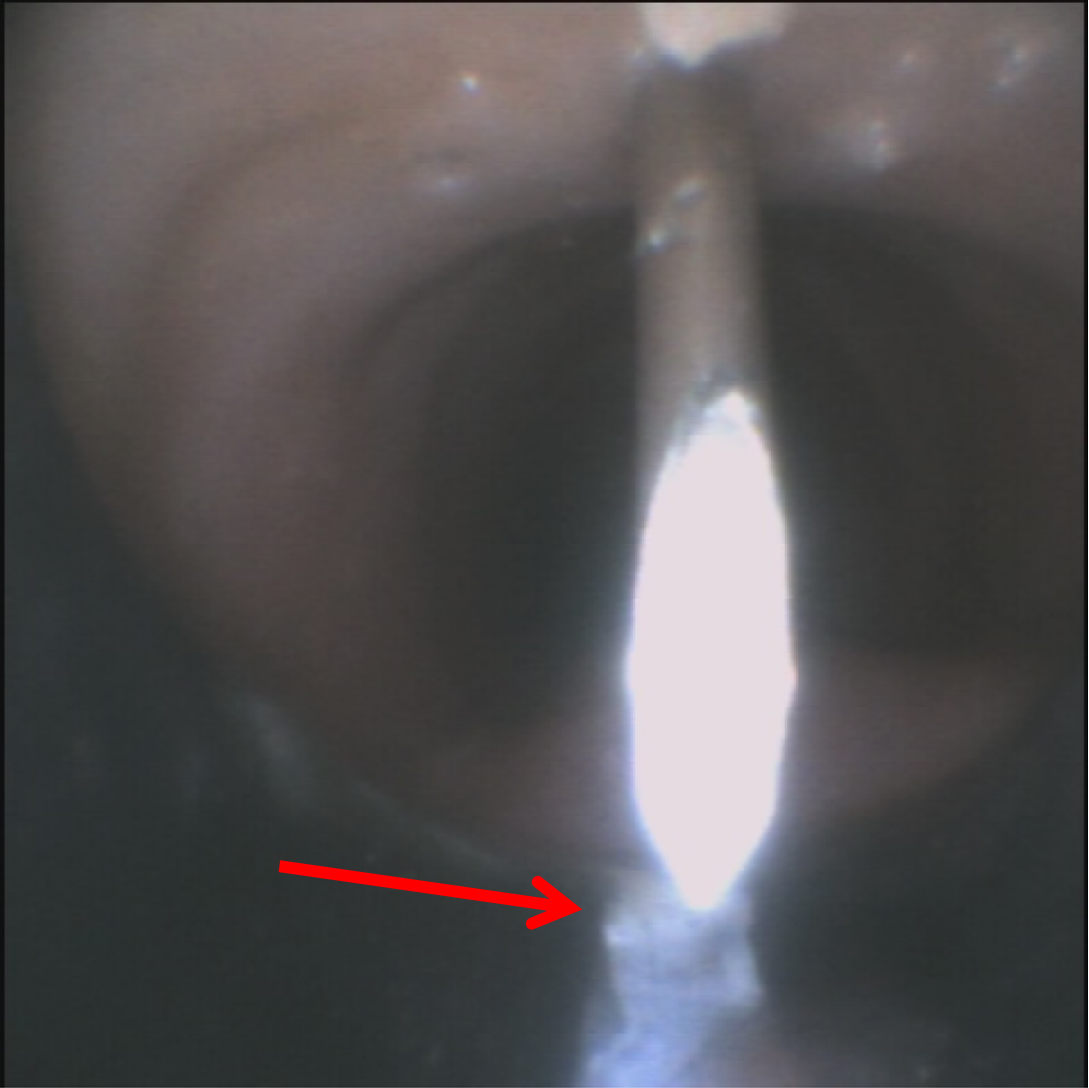
Endoscopic view of the needle. The short white line in the internal lumen is a marker positioned on the tracheoscope midline (180°) at the lower end (see red arrow)

*BMI* body mass index,

For all the endpoints, we found a significant difference in favour of tTNI over the standard method (Table 2). In particular, the number of attempts and the time needed were very low with tTNI, and there was significantly better precision in the placement of the puncture (Fig. 4).

**Table 2:** Comparison of translaryngeal Tracheostomy Needle Introducer versus standard needle puncture technique among all operators

Attempts by all operators (*N* = 58)	Average ± SD	Min	Max	Median	25th percentile	75th percentile	*p* value
Attempts with standard method (*N*)	1.91 ± 1.34	1	7	1	1	2	*p* < 0.001
Attempts with tTNI (*N*)	1.19 ± 0.5	1	3	1	1	1	
Time required for standard method (s)	36.8 ± 51.6	3	300	20	13	35.75	*p* < 0.001
Time required for tTNI (s)	13.14 ± 15.57	2	92	8	7	11	
Precision of first attempt* with standard method (°)	18.87 ± 25.38	0	120	15	0	30	*p* < 0.005
Precision of first attempt* with tTNI (°)	7.5 ± 12.95	0	60	0	0	15	

Data analysed using the Wilcoxon sign test

*Endoscopic reference: degrees from the anterior tracheal midline with the target ranging from − 30° to + 30°

*tTNI* translaryngeal Tracheostomy Needle Introducer

There were no anatomical or technical complications observed with tTNI use (Table 3).

**Table 3:** Complications of tracheal needle puncture in procedures by all operators

Complications	Standard (*N* = 58)		tTNI (*N* = 58)		Chi-square
	*n*	%	*n*	%	*p* value
Anatomical complications*					
Posterior tracheal wall puncture	6	10.3	0	0	0.0486 (*p* < 0.05)
Lateral tracheal wall puncture	3	5.2	0	0	0.2603
Final needle position outside the primary target (between the first and second tracheal rings)	5	8.6	0	0	0.0829
Technical complications^#^					
External puncture of ET	5	8.6	0	0	0.0829
ET cuff injury	1	1.72	0	0	0.9932

*Clinical complications: injury to anatomic structure or incorrect path of the needle

^#^Technical complications: damage to or incorrect use of technical equipment

*ET* endotracheal tube; *tTNI* translaryngeal Tracheostomy Needle Introducer

When we separately analysed the untrained operators, the number of attempts, time needed and precision of the punctures were still significantly better when they used the tTNI (Table 4).

**Table 4:** Comparison of translaryngeal Tracheostomy Needle Introducer versus standard needle puncture technique among untrained operators

Attempts by untrained operators (*N* = 32)	Average ± SD	Min	Max	Median	25th percentile	75th percentile	*p* value
Attempts with the standard method (*N*)	1.87 ± 1.53	1	7	1	1	2	*p* < 0.050
Attempts with the tTNI (*N*)	1.28 ± 0.5	1	3	1	1	1	
Time required for standard method (s)	46.8 ± 65.73	6	300	22.5	17	36.25	*p* < 0.001
Time required for tTNI (s)	17.21 ± 19.7	4	92	10	7.75	15.25	
Precision of the first attempt* with standard method	22.03 ± 30.47	0	120	15	0	30	*p* < 0.020
Precision of the first attempt* with the tTNI	5.16 ± 12.98	0	60	0	0	0	

*Endoscopic reference: degrees from the anterior tracheal midline with the target ranging from − 30° to + 30°

## Discussion

Our data show significant differences in favour of the use of tTNI over the standard method. In particular, we found a significant reduction in the number of attempts, time required (indicating the simplicity of the procedure) and greater precision of needle insertion. When we used the tTNI, we observed none of the complications that could be detected on the cadavers.

Another goal was to provide a device with a short learning curve that could be used by physicians with very different skill levels. A survey in Italy revealed that two thirds of ICUs had a specific tracheostomy team comprising several physicians and nurses trained in tracheostomy methods [9]. Our sub-analysis of the untrained operators also showed significant differences in favour of using the tTNI in terms of time needed and precision. The comparison of the number of attempts between the two procedures was just at the threshold of statistical significance (*p* = 0.048) (Table 4). The fact that no formal training in tTNI use was given to any operator prior to performing the procedure and that the sample size was relatively small might explain the failure to find a greater difference in the number of attempts required. Even if some minor technical improvements are still required, we believe that the tTNI can be a very useful tool even in ICUs without a specifically trained tracheostomy team.

Although PDTs are generally considered a routine ICU procedure, regardless of the particular technique preferred by the operator, the rate of complications is still far from zero [9, 10]. To our knowledge, there have been no studies directly comparing the safety of the commonly used techniques. At the same time, the heterogeneity in ICU practice and geographic spread of the techniques make it almost impossible to compare the incidence of complications reported in the different studies.

Our preference for Fantoni’s TLT method is primarily based on our view that extracting the cone cannula from inside the trachea to the outside to dilate the stoma minimises the risk of posterior tracheal wall injury, one of the most dangerous complications of PDT. Thus, stoma dilatation is directly performed using the final cannula, obviating the need to exchange the dilator for the final cannula. Pressure applied by the fingers on the neck skin during extraction of the cone cannula compresses the pre-tracheal tissues, minimising the risk of bleeding. Finally, throughout the TLT procedure, the patient’s airway is protected by the rigid tracheoscope, allowing mechanical ventilation and endoscopic vision. Thereafter, ventilation is accomplished through the long catheter of Fantoni’s kit.

We reviewed the existing devices for PDT, specifically developed for precise needle insertion into the trachea [11–14] (see Additional file 4: Table S1), but none of those were for use with TLT. The most interesting devices are from Margolin’s group [12, 13]. They developed devices for PDT based on a long Macintosh blade [12] or a metal stylet inserted in the endotracheal tube [13] to serve as an internal reference for an external arm that guides the needle into the trachea. SafeTrach™ [14] is the most recent and consists of a long, rigid metal stylet inserted in the endotracheal tube. The distal part of the stylet is plate-shaped and extends beyond the distal end of the endotracheal tube, protecting the posterior tracheal wall. The external arm, properly proportioned in length, is fitted onto the metal stylet. This device has produced very good results in tests on 17 patients, with higher precision of the puncture, lower complication rate and shorter time required for the PDT procedure [14].

However, in our opinion, the tTNI has several potential advantages, even over SafeTrach™. First, a rigid metal stylet in the tracheal lumen is not required. We consider it to be potentially dangerous, especially when it must protrude beyond the endotracheal tube. Such an approach may conflict with guidelines on orotracheal intubation [21]; moreover, there is a case report on tracheal injury secondary to distal protrusion of preloaded endotracheal tube stylet [22]. In our procedure, the posterior tracheal wall is directly protected by the ‘flute beak’ of the rigid tracheoscope. The tTNI itself is entirely external to the patient’s body, and the device always allows for continuous endoscopic monitoring of the procedure.

Finally, we emphasise on the low production cost of the device: we estimate the production of each single device to be approximately 200 euros, whereas for the production in series, we estimate the cost to be approximately 10 euros.

By experimenting on cadavers, which have real human anatomy and tissue consistency, we accumulated substantial technical information that will allow us to improve the device (e.g. modifying the fitting between the plastic support and the Plexiglas arm and better anatomic adaptation). Optimisation of the device and repeat testing on cadavers are important steps to take before testing it in human trials.

## Limitations of the study

Although our results with the standard method are in line with data in the literature, thus supporting the reliability of the experiment, there are some important limitations of the study, one being the small number of operators.

While testing the device on cadavers allowed for the assessment of certain complications, we could not evaluate the risk of bleeding, either minor (controllable by direct local pressure) or major (requiring surgical exploration). In addition, we could not simulate potential complications related to mechanical ventilation, such as pneumothorax, pneumomediastinum or subcutaneous emphysema.

## Conclusion

The tTNI is a safe device that simplifies needle insertion and reduces complications related to needle insertion during TLT. This study revealed that tTNI is safe in the hands of operators with a wide range of skill levels. A few minor technical improvements are necessary before conducting clinical trials of the device on patients. It is hoped that such trials will eventually confirm the safety and usefulness of the device.

## Additional files

Additional file 4: **Table S1.** Charracteristics of existing needle introducers for tracheal puncture. (DOCX 16 kb)

## Abbreviations

ICU: Intensive care unit
PDT: Percutaneous dilatational tracheostomy
TLT: Translaryngeal tracheostomy
tTNI: Translaryngeal Tracheostomy Needle Introducer

## References

[1] Abe T, Madotto F, Pham T, Nagata I, Uchida M, Tamiya N, Kurahashi K, Bellani G, Laffey JG, LUNG-SAFE Investigators and the ESICM Trials Group (2018). Epidemiology and patterns of tracheostomy practice in patients with acute respiratory distress syndrome in ICUs across 50 countries. Crit Care.

[2] Gi.Vi.Ti Group (2016). Progetto MargheritaPROSAFE–PROmoting patient SAFEty research and quality improvement in critical care medicine.

[3] Byhahn C, Wilke HJ, Halbig S, Lischke V, Westphal K (2000). Percutaneus tracheostomy: Ciaglia blue rhino versus the basic Ciaglia technique of percutaneous dilational tracheostomy. Anesth Analg.

[4] Westphal K, Maeser D, Scheifler G, Lischke V, Byhahn C (2003). PercuTwist: a new single-dilator technique for percutaneous tracheostomy. Anesth Analg.

[5] Fantoni A, Ripamonti D (1997). A non-derivative, non-surgical tracheostomy: the translaryngeal method. Intensive Care Med.

[6] Fantoni A, Ripamonti D, Lesmo A (1996). Translaringeal tracheostomy, a new era?. Minerva Anetesiol.

[7] Griggs WM, Worthley LI, Gilligan JE, Thomas PD, Myburg JA (1990). A simple percutaneous tracheostomy technique. Surg Gynecol Obstet.

[8] Araujo JB, Añón JM, García-Fernández AM (2015). Traqueotomía percutánea por dilatación con el método Ciaglia Blue Dolphin®. Med Int.

[9] Vargas M, Servillo G, Arditi E (2013). Tracheostomy in intensive care unit: a national survey in Italy. Minerva Anestesiol.

[10] Fernandez-Bussy S, Mahjan B, Folck E, Caviedes I, Guerrero J, Majid A (2015). Tracheostomy tube placement: early and late complications. J Bronchol Intervent Pulmonol.

[11] Dauri M, Improta S (2007). Device for tracheotomy WO 2007/017447 A2, 15 feb 2007.

[12] Margolin G, Karling J (2011). A dilator assembly, a device to facilitating tracheostomy and method of making a percoutaneus tracheostoma WO 2011/012554 A1, 3 feb 2011.

[13] Margolin G, Karling J (2007). Device and method for tracheotomy WO 2007/018472 A1, 15 feb 2007.

[14] Ullman J, Karling J, Margolin G (2016). A new safe and cost-effective percutaneous dilatational tracheotomy: SafeTrach. Acta Otolaryngol.

[15] Leung KS, Taglang G, Bühren V, Sato K, Born CT, Probe R, Vécsei V, Maxey J (2014). Gamma3® Long Nail R1.5 and R2.0.

[16] Opendot Lab, Milan (2018) http://www.opendotlab.it. Accessed 15 May 2018.

[17] Hackability Project (2017) http://www.hackability.it. Accessed 15 May 2018.

[18] Balboni G, Anastasi G (2010). Anatomia Umana, Trattato.

[19] Eisma R, Lamb C, Soames RW (2013). From formalin to thiel embalming: what changes? One anatomy department’s experiences. Clin Anat.

[20] Eisma R, Gueorguieva M, Immel E, Toomey R, McLeod G, Soames R, Melzer A (2013). Liver displacement during ventilation in Thiel embalmed human cadavers–a possible model for research and training in minimally invasive therapies. Minim Invasive Ther.

[21] Kabrhel C, Thomsen TW, Setnik RM (2007). Videos in clinical medicine. Orotracheal intubation. N Engl J Med.

[22] Warner MA, Fox JF (2016). Direct laryngoscopy and endotracheal intubation complicated by anterior tracheal laceration secondary to protrusion of preloaded endotracheal tube stylet. Clin Care.

