# Supplementary material for: Translaryngeal Tracheostomy Needle Introducer: a simple device to improve safety and reduce complications during Fantoni’s translaryngeal tracheostomy procedure: trial on human cadavers
Source: Intensive Care Med Exp. 2019 Jan 28;7:9. doi: 10.1186/s40635-019-0221-x (PMC6890899; doi:10.1186/s40635-019-0221-x)
Supplement: Supplementary file 4 — Table S1. Charracteristics of existing needle introducers for tracheal puncture. (DOCX 16 kb) [file 40635_2019_221_MOESM4_ESM.docx]

**Table S1:** Characteristics of existing needle introducers for tracheal puncture

| **Device**  **Name** | **Authors** | **Patent** | **Internal Tracheal Reference** | **External Arm** | **Needle**  **Insertion**  **Inclination** | **Protection of Posterior Tracheal Wall** | **Endoscopic**  **View of the Procedure** |
| --- | --- | --- | --- | --- | --- | --- | --- |
| Device for Tracheotomy | Dauri M, Improta S [10] | WO 2007/017447 | Special ET | Mobile | Variable | Special long ‘flute beak’ | Possible |
| A dilator assembly, a device for facilitating tracheostomy and methods of making a percutaneous tracheostoma | Margolin G,  Karling J [11] | WO 2007/018472  WO 2010/0500875  WO 2011/012554 | Very long Macintosh blade | Rigid | 90° | Terminal part of long Macintosh blade | Not easy |
| Device and method for tracheostomy | Margolin G,  Karling J [12] |  | Curve metal stylet | Mobile | 90° | Scoop-shaped device outside the ET | Not easy |
| Safe Trach™ | Ullman J  Margolin G,  Karling J [13] |  | Long and straight metal stylet | Long and fixed | <90°  (from top to bottom) | Plate- shaped device outside the ET | Possible with thin endoscope |
| Translaringeal Tracheostomy Needle Introducer | Terrani A, Bassi E,  Foti G | Italian Patent pending n° 102017000035827 | Rigid tracheoscope Fantoni’s Kit | Fitted on tracheoscope with a dedicated support | >90°  (from bottom to the top) | ‘Flute beak’ of rigid tracheoscope | Possible |

ET: endotracheal tube

We searched of both the Pubmed medical database and a technical patent database (<https://worldwide.espacenet.com/advancedSearch?locale=en_EP>) for existing devices specifically developped for precise needle insertion in the trachea with the following keywords:

“tracheostomy, percutaneous, introducer, needle”
